# Utilization of Modern Contraceptives among HIV Positive Reproductive Age Women in Tigray, Ethiopia: A Cross Sectional Study

**DOI:** 10.1155/2013/319724

**Published:** 2013-09-25

**Authors:** Yemane Berhane, Haftu Berhe, Gerezgiher Buruh Abera, Hailemariam Berhe

**Affiliations:** ^1^Axum College of Nursing, Tigray, Ethiopia; ^2^Department of Nursing, College of Health Sciences, Mekelle University, Ethiopia

## Abstract

*Background*. HIV infected women in sub-Saharan Africa are at substantial risk of unintended pregnancy and sexually transmitted infections. In developing countries including Ethiopia counseling and provision of modern contraceptives of choice to HIV infected women including those on antiretroviral therapy (ART) is an important strategy to prevent unintended pregnancies and sexually transmitted infections. Little is known about the existing practices and utilization of modern contraceptives among HIV positive reproductive age women attending ART units. *Objective*. The aim of this study was to assess utilization of modern contraceptives and associated factors among HIV positive reproductive age women attending ART units in zonal hospitals of Tigray region, North Ethiopia. *Method*. Institution based cross-sectional study was conducted by interviewing 364 HIV positive reproductive age women in all zonal hospitals of Tigray region using systematic sampling technique. Structured and pretested questionnaire was used to obtain information from the respondents. Descriptive, bivariate, and multivariate methods were used to analyze utilization of modern contraceptives and the factors associated with it. *Result*. Three hundred sixty-four subjects participated with a response rate of 99.2%. The mean age of the respondents was 31.9 ± 6.5 (SD) years. About 46% of participants utilized modern contraceptives, 59.9% out of them used dual method. However, a significant proportion of the respondents (46%) reported that they wished to have a desire for children. Being secondary education and higher (AOR: 2.85; 95% CI: 1.17–6.95) and currently on HAART (AOR: 3.23; 95% CI: 1.49–7.01) they were more likely to utilize modern contraceptive. But those women who were ≥25 years old, house wives, single, divorced, or widowed were less likely to utilize modern contraceptive. *Conclusion*. Results of this study revealed that the number of respondents who were ever heard of modern contraceptives was high. However, modern contraceptive utilization was still low. Additional efforts are needed to promote modern contraceptive utilization in general and dual method use in particular among HIV positive reproductive age women.

## 1. Introduction

At the Alma-Ata conference (1978), Family Planning services were highlighted as one of the basic and important strategies for reducing high risk pregnancies that often occurred too early, too late, and too frequent and also as a way to improve child heath. Family planning programs have helped women worldwide to avoid millions of unintended pregnancies often associated with high risk abortions since the 1960s [1].

In many countries, PMTCT programs focus on antenatal HIV testing and provision of ARV prophylaxis to HIV infected women and their newborns. However, these three components constitute just one of the four pillars for PMTCT, the remaining being primary HIV prevention in women of child-bearing age, family planning (FP) for the prevention of unwanted pregnancies, and care and treatment for HIV infected women and their HIV affected children. HIV/AIDS has had devastating effects and is currently a common complication of pregnancy in Ethiopia [2].

Counseling and provision of contraception of choice to HIV infected women including those on ART is an important strategy to prevent unintended pregnancies among HIV positive women who would rather wish to delay pregnancy or stop child bearing altogether. Dual function contraceptives that simultaneously prevent HIV transmission as well as unwanted pregnancies might be the most appropriate contraceptive method for women living with HIV/AIDS [3].

Contraception can be provided either within the treatment programs or by active referral to routine family planning providers. This would reduce number of pregnancies among HIV positive women and reduce the number of HIV infected infants. The other benefit of contraception is that it averts HIV infection in infants at a lower cost compared to other PMTCT interventions. Indeed the prevention of unintended pregnancies among women living with HIV is one of the World Health Organization (WHO) four elements of comprehensive approach to prevent mother-to-child transmission of HIV [4–7]. 

The Millennium Development Goals, adopted in New York in 2000, promote universal education and gender equality, maternal and child health, and prevention and treatment for HIV/AIDS [8]. 

Provision of comprehensive reproductive health care is central to attaining these goals Worldwide, as many as one-third of the 357,000 annual maternal deaths are attributable to unintended pregnancies; the majority of these mortalities occur in low- and middle-income countries [9–11]. Enhanced access to family planning services particularly modern contraceptives, in sub-Saharan Africa, would result in marked reductions in unintended pregnancies and unsafe abortions, a projected 69% decrease in maternal deaths, and a 57% decrease in newborn deaths [10].

 In addition to substantial risks of dying from pregnancy complications, women in sub-Saharan Africa are also at increased risk of HIV infections. Providing safe, effective contraception to HIV infected women who desire it has also been identified by the World Health Organization as a primary strategy for prevention of pediatric infections [12]. 

Ethiopia is one of the countries most severely affected by HIV/AIDS pandemic. According to the 2007 single point HIV prevalence estimate of Ethiopia for the year 2010, the average adult female HIV prevalence was 2.9%. Out of the total HIV positive populations in the country, 717,669 were females, of which 90,311 HIV positive pregnant women. For Tigray according to 2007 single point HIV prevalence estimate, the total adult female HIV prevalence for 2010 was 3.7%. There were 7,073 HIV positive pregnant women [2, 12, 13]. Utilization of modern contraceptives among HIV positive reproductive age women ever enrolled in ART units, in Tigray region, is among those essential and critical issues which need study in wide range, but little is known about. 

## 2. Methodology

### 2.1. Setting

The study was carried out in all zonal hospitals of ART units in Tigray region. Tigray region is the Northern most National Regional state of Ethiopia. The region covers about 50,000 square kilometers. The total population of Tigray projected based on 2007 statistical census agency is about 4.3 million of which 82.6% are rural and 51% are female. According to the 2003 (EFY) Health and Health Related Indicators published by FMOH, Tigray has 14 hospitals (1 referral, 6 zonal, and 7 district hospitals), 209 health centers, and 572 health posts that give health service coverage of 83% [14]. Currently there are about 6500 HIV positive women enrolled in the ART units of the zonal hospitals. An institution based cross-sectional study design was conducted, and it was conducted from May to December, 2012.

The source population was all HIV positive women of reproductive age (15–49 years) attending ART units, and the study population was sampled HIV positive women of reproductive age (15–49 years) attending ART clinics. 

The sample from each hospital was allocated proportionally to the number of clients on ART at each institution. The sample size for the study was determined by assuming prevalence of modern contraceptive utilization (*P*) to be 59% from the study conducted in Lusaka, Zambia among HIV infected women [15], a confidence level of 95% (CI), and marginal error of 5% (*d*) giving a sample size of 372.

Based on evidences obtained from the respective zonal hospitals, the source population was 2970. So population correction was used to get the corrected finite sample size that was 331. To compensate for nonresponse rate 10% of the sample was added =33; finally a total of 364 HIV positive women were sampled for the study. The total sample size was proportionally allocated to all zonal hospitals based on the number of client flow rate on 3 weeks prior to the study. Systematic random sampling technique was used to select eligible participants from each hospital ART units. 

Structured and pretested questionnaire was used to obtain information from the respondents. The tool consists of demographic, socioeconomic characteristics, information on contraceptive utilization, and associated factors. An exit interview was conducted during the regular working hours by the trained nurses. We also had conducted a medical record review to confirm HAART history and to obtain clinical data (WHO stage of disease and CD4 cell count). Six degree holder nurses were supervising the data collection in the respective hospitals. 

The dependent variable was utilization of modern contraceptives, and the independent variables were demographic characteristics, fertility related factors, socio-economic factors, HIV related factors, duration on ART and ART status, drug side effects, contraceptive service integration, WHO clinical stage of disease, disclosure, and partners HIV status.

*Contraceptive utilization*: sexually active respondents who responded positively after being asked whether they utilized modern contraceptive in the last one month. 

*Modern methods*: including pills, IUD, injectables, implants and male and female condoms (excluding lactational amenorrhoea, rhythm, and withdrawal). 

*Consistent condom use*: use of male or female condom during every sexual intercourse. 

*Dual contraceptive methods*: utilization of any hormonal or permanent modern contraceptive method along with male or female condom. 

*Integration of contraceptive services*: referred to a report from participants who received a contraceptive method in a spot with the ART services. 

To maintain the data quality, the data collectors and supervisors were trained in the methods, objectives, confidentiality, and other technical aspects of the study before data collection commenced. The questionnaire was first prepared in English, translated into the local language (Tigrigna), and then translated back to English. The questionnaire was pretested in 5 percent of the participants in Wukro hospital a week before the commencement of the main research. Completed questionnaire was cross-checked daily for inconsistencies and completeness.

The data was coded, entered, edited, and analyzed using SPSS version 16.0 for windows. Binary logistic regression was used to test the significance of associations. Important associations (odds ratio, confidence intervals, and *P* value) were also calculated furthermore, multi-co-linearity diagnostics and interaction of variables were tested by the use of logistic regression model.

Ethical clearance and approval was obtained from Mekelle University College of the Health Sciences; Permission was also secured from Tigray Regional Health Bureau and from the six zonal hospitals. Participation was on a voluntary basis after informed verbal consent, and responses were kept confidential.

## 3. Result

A total of 364 subjects participated with a response rate of 99.2%. Overall, 315 (87.3%) of the respondents were from the urban area. The mean age of the respondents was 31.9 ± 6.5 (SD) years. Approximately, 55 percent of the participants were in the age group of 25 to 34 years. Three hundred thirty-two (92%) were from orthodox religion and 338 (93.6%) were ethnically Tigrians. In terms of educational status, 87 (24.2%) were illiterate. Over 43% were currently married, and the majority (71.5%) had primary level of education or less. Over half, 195 (54%), were unemployed, and the majority of the respondents, 171 (47.40%), reported earning less than or equal to 500 ETB a month (Table 1). 

Results of this study revealed that 100% of the study subjects ever heard at least one modern contraceptive. Three hundred sixty one women have practiced sex-one month prior to data collection. Less than half, 167 (46.3%), were currently using a modern contraceptive method. The most commonly used method, 100 (59.9%), was dual contraceptives. Overall, 29.3% of the respondents reported using hormonal methods only (injectables, pill, and Norplant; 22.7%, 4.2%, and 2.4% resp.). IUDs were not reported by any respondent. Out of the dual or condom only users 109 (92.4%) reported utilizing condom consistently. Current use of modern FP methods was the highest, 68 (40.7%) among the women with secondary and above level education. In this study married women were found to be using modern contraceptives the most 110 (65.9%). 

The main reason women choose current method was health professionals advice, 54 (32.3%), easy to use, 44 (26.3%), and perceived less side effects, 29 (17.4%). 

About half of modern contraceptive users (49.7%) got the service within the ART clinic. A substantial number of women, 194 (53.7%), were not currently utilizing any modern method. Among those who did not use modern contraceptives, 95 (49%) stated their reason as fear of drug interaction. However, 235 (65.1%) of the participants were intended to continue or use in the future (Table 2).

Overall, it was found that 166 (46%) of all participants wanted to have more children, and considerable proportion of women (79.8%) have at least one child. Out of the women that were using modern contraceptives, 49.1% had 2-3 children followed by one or no living children. Among the women who did not desire having more children, 56.3% (94) were currently using modern methods. With regards to their partner's serostatus, 161 (85.5%) were aware of their partner's serostatus (153 (82.3%) seropositive and 6 (3.2%) seronegative). The rest, 27 (14.5%) did not know about their partner's HIV status. The median time since first HIV diagnosis was 3.00 years, ranging from 0.1 to 9.9 years. More than half (68.7%) had recent CD4 counts >250 cells/mm^3^ (mean recent CD4 = 376.8 (SD = 147.7)) with the mean duration on ART of 3.4 years ± 1.86 (SD). Nearly 60% of women were in WHO stage of disease I or II and 92% had disclosed their HIV status to their regular partner. Two hundred and twenty-four (81.4%) participants were currently on HAART while 67 (18.6%) were not. One hundred forty-four (86.2%) of women on HAART utilize modern methods. A large number of participants 212 (58.7%) had been concerned about side effects of method choice like irregular bleeding, infertility and vomiting. 41% of those who have no or only one child had utilized modern contraceptives (Table 3).

In Bivariate analysis, women were less likely to report modern contraceptive use if house wives, single, divorced, or widowed. Conversely, secondary or higher education attended, and those who had a monthly income of 1501–2500 ETB, being counseled for contraceptive, those with a CD4 count ≤250 cells/mm^3^ and currently on HAART had increased odds of contraceptive use. The results from the multivariable logistic regression modeling revealed that independent predictors of modern contraceptive utilization for HIV positive women of reproductive age in zonal hospitals, Tigray region, were age, occupation, educational status, and currently on HAART. Therefore, women 25–34 years old (AOR: 0.30; 95% CI: 0.12–0.79), ≥35 years old (AOR: 0.31; 95% CI: 0.11–0.87), and house wives (AOR: 0.4; 95% CI: 0.17–0.93) had lower odds of using modern contraception. The odds of using modern contraception were higher among women with secondary education and higher attended (AOR: 2.85; 95% CI: 1.17–6.95). Similarly, those women currently on HAART were 3.23 times more likely to utilize modern methods (AOR: 3.23; 95% CI: 1.49–7.01). There were no significant independent associations between contraceptive use and counseling for contraceptive, house hold monthly income, and CD4 cell count in adjusted analyses (Table 4). 

## 4. Discussion

This study attempted to assess utilization of modern contraceptives and associated factors among HIV positive reproductive age women. Overall, less than half (46.3%) of HIV positive reproductive age women in this study (86.2% ART experienced and 13.8% pre-ART) reported modern contraceptive use which was higher than the modern contraceptive prevalence rate in Tigray region irrespective of HIV status (21.2%) [16]. The higher uptake of family planning among this population might be due to high injectables and condom use rates and due to time deference. But, still congruent with the national target for CPR in the general population (44%) to achieve MDGs by 2015 though the prevalence of modern contraceptive in HIV positive women is expected to become higher than the general population to avert vertical and horizontal transmission of the virus [17]. Similarly, our finding was higher than that of the study done in Addis Ababa (28%) [18] and Rwanda (31%) [19], but in line with the report on contraceptive utilization in Malawi (46%) [20]. In contrary, the study has found that current use of modern contraceptive methods was lower than that from findings of a study done in Asela hospital, Oromia, Ethiopia, which is estimated at 76.5% [21]. This variation might be due to high desire for children, 46% in this study. The current finding is also lower than findings from Mbarara, Uganda (85%) [22], Lesotho (82–86%) [23], Lusaka, Zambia (59.2%) [20]. The possible reason for this might be due to geographical variation. 

Even though modern contraceptive methods were widely available at free cost in ART treatment units and/or hospitals, more than half were not using any modern methods. While the Ethiopian AIDS Control Program National guidelines advocate for dual family planning methods to prevent HIV/STI transmission and unintended pregnancies for HIV positive individuals, only (59.9%) women reported dual method use in this study. But this finding was higher than that of the study finding in Lusaka, Zambia [20]. The possible difference might be due to geographic and time variations and study design used. 

Out of the dual method/condom users, about 70% of women utilize condom consistently which is broadly in line with a study done in Soweto, South Africa [24]. Use of only hormonal contraceptive was found to be low (Depo provera (22.7%) followed by pills (4.2%) and only 2.4% reported use of Norplant). IUDs only were not reported by any respondent. This finding is congruent with EDHS report in Tigray region, irrespective of HIV status [25]. Similar results were also found in a survey done in Uganda [21]. 

In the study area, the most common reason for method choice was health professional's advice (32.3%). This finding is consistent with some studies elsewhere in Ethiopia [25, 26].

The study revealed that considerable proportion of women respondents (65.1%) have intention to use or continue to use modern contraceptive utilization. This finding is higher than that of the study done in Gondar university hospital [27]. This might be related to the fact that large numbers of women attending ART units have improved their awareness through time on contraceptive utilization due to frequent counseling services. 

To date, published experiences and data on provision of contraception services within HIV care clinics were limited. Yet this remains one of the most promising options for providing family planning for HIV positive women and deserves particular attention. This study showed that among those currently using a contraceptive method, 98.8% of women had been counseled on family planning methods and only 49.7% were provided at the antiretroviral treatment clinic. Though the finding is higher than that of a study done in Mombasa, Kenya [28], this entails the need to integrate family planning and other reproductive health services with the general ART service and may be one of the problems to meet reproductive health care needs of these women. Service integration offers options for preventing unintended pregnancies to HIV positive clients (using/not using ART), and as a part of PMTCT services.

Access to ARV is increasing in developing countries and enables HIV positive individuals to live longer and healthier lives. For instance, studies from industrialized countries suggest that ARV is associated with changes towards unsafe sex [29]. However, results from developing nations have been mixed [30, 31]. Conversely, increased contact with health care professionals to receive treatment may encourage positive changes in sexual behavior. This study revealed that women receiving HAART were 3.23 times more likely to report contraceptive use than pre-ART women. Our finding, indicating significantly higher prevalence of contraceptive use among HAART users compared with pre-ART women is broadly consistent with recent findings from Uganda, Mbarara and Soweto, South Africa [18, 22]. The reasons for higher contraceptive prevalence among HAART users were not directly explored in this study. However, an important possible reason for the observed differences is that women receiving HAART have more regular contact with health care professionals as a function of the clinical follow-up. During these regular clinic visits, reproductive and sexual health issues are raised, and the opportunity to discuss and commence use of contraception is presented. In general, ART provides opportunity to discuss FP and other RH matters. Hence receiving ART was associated with increased contraceptive utilization among the study participants which may indirectly informs us about the effectiveness of the HIV prevention program in the hospitals. In contrast to this, a study in Ghana showed that participants receiving HAART were significantly less likely to utilize contraceptives [32]. 

Although not statistically significant, half of the women who have concern about side effects of methods were not utilizing contraceptives. Studies elsewhere in Africa showed that concerns about side effects and inconveniences were by far the most prominent reasons for low utilization or discontinuation of modern contraceptives among women [33, 34].

HIV status disclosure to regular partner was not associated with use of contraception in either Bivariate or multivariable analyses. This is in contrast to a recent study in Uganda that showed that the lack of HIV disclosure was associated with lower odds of use of modern contraceptives among HIV women enrolled in HIV clinics in Uganda [35]. 

Sociodemographic factors (age, education, and occupation) were associated with contraception use. This study noted that women attending secondary education and higher had increased odds of contraceptive use, similar to findings reported from some African countries [33–36]. The higher the level of education, the more they appreciate the advantages of contraceptive use compared to those without education. The possible reason might be in this study is that most secondary education and higher attended women were married (currently with their regular partner) so frequent sexual contact may predispose them to unintended pregnancy and STIs. Individuals with more education might have higher levels of HIV/AIDS knowledge and are less likely to have stigma towards HIV/AIDS, thus enabling them to easily change risky sexual behavior. Additionally, individuals with higher education might be able to seek and accept information that would help protect their partners and themselves from HIV and STI transmission and re-infection. This finding is in line with the Ethiopian family planning guideline report which declared that women education increases FP use, improves communication with partner, and advance women's status in the community [16].

 Younger age was associated with increased contraceptive use, though the desire for future children is high in this age group. This finding is in line with study done in Mbarara, Uganda and Zambia, Lusaka [20, 22]. House wives were less likely to utilize modern contraceptive as compared to government employees in this study. Similar findings were found from a study done in Southern Nation Nationalities and People's Region, Mexico, and Ghana [36]. This might be due to the effect of husband's reproductive health decision making power that could overwhelm the effect of women's financial and domestic decision making autonomy on couple's family planning use. On the other hand, a significant proportion of the respondents (46%) reported that they wished to have a desire for children which is in line with a study done in Addis Ababa (44.7%) [26].

## 5. Limitations of the Study

- First, the cross-sectional study design makes it difficult to determine the direction of causality.
- Second, there is a risk of social desirability bias whereby HIV positive women may over-report their contraceptive use because of pressure from health workers and community members to practice protected sex.

## 6. Conclusion

Modern contraceptive method heard among the participants in this study population is high; however, modern contraceptive utilization was still low. Dual method use was about 60%. Use of certain long-term contraceptive methods available in the hospitals, such as implants and IUDs, was low. 

Likelihood (chances) of being current user of modern contraceptives by a woman increased with the increase in education level, being daily laborer, being government employee, and ART experience/being on HAART. Old age was associated with reduction in odds (chances) of being current user of modern contraceptives. High number of current family planning users and some others intended future modern contraceptive use. Moreover, a significant proportion of the respondents (46%) reported that they wished to have a desire for children.

- As countries plan improvement strategies to enhance contraception uptake among HIV infected reproductive age women, additional efforts are needed to promote modern contraceptive utilization in general and dual method use in particular.
- Investing in women education better to be strengthened.
- Regional health bureau should consider resource mobilization and address issues of demand together with stakeholders for promoting and strengthening the FP and other reproductive health issues.
- Health care providers who are assigned in ART clinics should consider implications for preventing vertical and heterosexual transmission of HIV, the need for appropriate counseling for contraceptives, and the importance of HARRT use.
- Hospital managers and clinical officers need to consider integration of family planning with ART services.
- Further exploration (magnitude of unwanted pregnancy) among HIV positive reproductive age women is recommended.

## Figures and Tables

**Table 1:** Sociodemographic characteristics of HIV positive reproductive age women attending ART units (*n* = 361), zonal hospitals, Tigray, North Ethiopia, 2012.

Variables	Frequency (*N*)	Percentage (%)
Age		
15–24	36	10
25–34	197	54.5
≥35	128	35.5
Education		
Illiterate	87	24.2
Read and write	85	23.5
Primary (1–8)	86	23.8
Secondary education (9 and above)	103	28.5
Religion		
Orthodox	332	92
Muslim	22	6.1
Others	7	2
Marital status		
Married	156	43.2
Single	33	9.1
Divorced/separated	121	33.5
Widowed	51	14.2
Occupation		
House wife	111	30.7
Daily laborer	84	23.3
Government employee	101	28
Private employee	65	18
Monthly house hold income		
0–500	171	47.4
501–1500	140	38.8
1501–2500	35	9.7
≥2501	15	4.1
Residence		
Urban	315	87.3
Rural	46	12.7

**Table 2:** Current utilization of modern contraceptives among HIV positive reproductive age women attending ART units, zonal hospitals, Tigray, North Ethiopia, 2012.

Variables	Frequency (*N*)	Percentage (%)
Ever heard of modern Contraceptives (*N* = 361)		
Yes	361	100
No	0	0
Total	**361**	**100**
Type of method ever heard		
Pill	43	11.9
Injectables	11	5.3
Male condom only	05	1.4
Dual methods	78	21.6
Any greater than or equal two methods	216	59.8
Total	**361**	**100**
Reason to choose method (*N* = 167)		
Health professionals advice	54	32.3
Perceived less side effects	29	17.4
Observed friends experience	18	10.8
Easy to use	44	26.3
Agreement with partner	2	1.2
Effectiveness	20	12
** **Total	**167**	**100**
Reasons for not to use contraceptives (*N* = 194)		
Fear of drug interaction	95	26.3%
Trying pregnancy	78	21.6%
Partner does not want contraceptive	18	5%
Others	3	0.8%
Total	**194**	**53.7%**

**Table 3:** Fertility, HIV, and clinical related factors among HIV positive reproductive age women, zonal hospital, Tigray, North Ethiopia, 2012.

Variables	Modern contraceptive utilization	
	Yes (%)	No (%)
Desire for children		
Yes	73 (43.7%)	93 (47.9%)
No	94 (56.3%)	101 (52.1%)
Partner's serostatus (*N* = 186)		
Negative	6 (4.2%)	0
Positive	117 (83%)	36 (80%)
Unknown	18 (12.8%)	9 (20%)
Discuss with partner about contraceptive (*N* = 186)		
Yes	133 (94.3%)	42 (93.3%)
No	8 (5.7%)	3 (6.7%)
HIV disclosure to regular partner		
Yes	127 (90.1%)	44 (97.8%)
No	14 (9.9%)	1 (2.2%)
Side effects (*N* = 212)		
Irregular bleeding	63 (60.6%)	67 (62%)
Vomiting	12 (11.6%)	11 (10.2%)
Infertility	25 (24%)	26 (24.1%)
Others	4 (3.8%)	4 (3.7%)
Counseling service for contraceptive		
Yes	165 (98.8%)	183 (94.3%)
No	2 (1.2%)	11 (5.7%)
Current HAART use		
Yes	144 (86.2%)	150 (77.3%)
No	23 (13.8%)	44 (22.7%)
Years since on ART2		
0.1–2.0 years	29 (20.1%)	57 (38%)
≥2.1 years	115 (79.9%)	93 (62%)
WHO clinical stage		
1/2	99 (59.3%)	117 (60.3%)
3/4	68 (40.7%)	77 (39.7%)

**Table 4:** Bivariate and multivariate analyses of variables associated with contraceptive use among HIV positive women (aged 15–49 years), attending ART units in zonal hospitals, Tigray, (*N* = 361) North Ethiopia, 2012.

Variable	Utilize contraceptive		Crude OR (95% CI)	Adjusted OR (95% CI)	*P*-value
	Yes (%)	No (%)			
Educational status					
Illiterate	29 (17.4%)	58 (29.9%)	1	1	
Read and write	39 (23.3%)	46 (23.7%)	1.70 (0.92, 3.14)	1.45 (0.66, 3.15)	
Primary (1–8)	31 (18.6%)	55 (28.4%)	1.13 (0.60, 2.11)	0.92 (0.41, 2.09)	
Secondary and above (grade 9 and above)	68 (40.7%)	35 (18.0%)	3.89 (2.12, 7.11)*	2.85 (1.17, 6.95)**	0.02
Occupation 2					
Government employee	66 (39.5%)	35 (18.0%)	1	1	
Daily laborer	35 (21%)	49 (25.3%)	0.38 (0.21, 0.69)*	0.98 (0.43, 2.27)	
House wives	38 (23.8%)	73 (37.6%)	0.28 (0.16, 0.49)*	0.4 (0.17, 0.93)**	0.034
Private employee	28 (16.8%)	37 (19.1%)	0.40 (0.21, 0.76)*	0.51 (0.23, 1.12)	
Age group (years)					
15–24	19 (11.4%)	17 (8.8%)	1	1	
25–34	97 (58.1%)	100 (51.5%)	0.87 (0.43, 1.77)	0.3 (0.12, 0.79)**	0.015
≥35	51 (30.5%)	77 (39.7%)	0.59 (0.28, 1.2)	0.31 (0.11, 0.87)**	0.026
Grouped monthly income (ETB)					
≤500	67 (40.1%)	104 (53.6%)	1	1	
501–1500	69 (41.3%)	71 (36.6%)	1.5 (0.96, 2.37)*	1.38 (0.77, 2.48)	
1501–2500	22 (13.2%)	13 (6.7%)	2.6 (1.24, 5.57)*	1.98 (0.76, 5.19)	
≥2501	9 (5.4%)	6 (3.1%)	2.3 (0.79, 6.84)*	2.43 (0.66, 8.96)	
Counseling service for contraceptive					
Yes	165 (47.4%)	183 (52.6%)	4.96 (1.08, 22.7)*	5.07 (0.71, 36.1)	
No	2 (15.4%)	11 (84.6%)	1	1	
Grouped CD4					
≤250 cells/mm^3^	63 (37.7%)	50 (25.8%)	1.74 (1.1, 2.73)*	0.94 (0.50, 1.75)	
>250 cells/mm^3^	104 (62.3%)	144 (74.2%)	1	1	
Current HAART use					
Yes	144 (86.2%)	150 (77.3%)	1.84 (1.06, 3.20)*	3.23 (1.49, 7.01)**	0.003
No	23 (13.8%)	44 (22.7%)	1	1	

*Statistical significance in COR, **statistical significance in AOR, OR: odds ratio, CI: confidence interval, and 1: reference.
